# Prediction of Fractures in Coal Seams with Multi-component Seismic Data

**DOI:** 10.1038/s41598-019-42956-7

**Published:** 2019-04-24

**Authors:** Mengqi Li, Jun Lu, Shu Xiong

**Affiliations:** School of Energy Resources, China University of Geosciences, Beijing, 100083 China

**Keywords:** Geophysics, Seismology

## Abstract

Fractures that develop in coal seams threaten safety in many ways, but they can be predicted using fracture parameters derived from seismic data. However, the post-stack split shear waves are difficult to thoroughly separate by Alford rotation due to wavefield mixing. We propose a method of predicting fractures in a coal seam using multi-component seismic data, which was applied to coal seam 13-1 of the Huainan coalfield, China. We employed the Alford rotation to separate the split PS-waves (P-to-S converted waves) and perform interlayer travel-time inversion of the fast shear waves using geophysical logs, rock-physics parameters, and tunnel-excavation information as constraints. However, post-stack wavefield mixing of the coal seam interfered with the Alford rotation of the real post-stack seismic data. Therefore, we only performed the Alford rotation on radial and transverse component post-stack sections to derive fracture azimuths, which were then applied to the pre-stack separation of the split PS-waves. Using joint PP- and PS-wave inversion, anisotropy parameters were derived for use in fracture prediction. Finally, we predicted unsafe mining areas with a high probability of coal and gas outbursts. The application results were verified by excavation data from the mine tunnels. Our method contributes to fracture prediction on coal mine safety.

## Introduction

The phenomenon of shear wave (S-wave) splitting was discovered in the study of crustal anisotropy^1,2^. In fractured media, when the S-wave polarization direction is oblique to the fracture plane, it will be split into a fast shear wave (S1-wave) and a slow shear wave (S2-wave) polarized parallel and orthogonally to the fracture plane, respectively. There is a time delay between the S1- and S2-waves due to the difference in propagation velocities, which indicate the degree of fracture development. Thus, using the phenomenon of S-wave splitting to predict the development of fractures is an important geoscience research topic.

The separation of split S-waves has been applied in many studies based on multi-component seismic data. For example, the technique can help deduce the anisotropy of the crust and upper mantle to estimate crustal deformation, the flow of deep materials, and the preferential orientation of mineral alignment^3,4^. For strata with multiple sets of fractures, layer stripping and compensation of PS-wave (P-to-S converted waves) splitting can improve the quality of PS-wave images^5,6^. Fractures are necessary for the cost-effective development of unconventional gas resources, such as tight sandstone gas, shale gas, and coalbed methane^7,8^. However, in the context of coalfield production, fractures are gas storage spaces, which pose risks of coal and gas outbursts. Hence, fracture prediction based on split S-wave analysis is useful for both the exploration and exploitation of unconventional reservoirs and the improvement of coal mine safety.

The relation between coal and gas outbursts and the development of fractures has been proven^9^. Due to the formation pressure and strong adsorption capacity of coal, the fractures and micropores of coal seams are enriched by significant quantities of gas^8–10^. When the local formation pressure is released by excavation, the risk of coal and gas outbursts will increase due to the sudden transition of gas from the adsorbed state to the free state^11^. According to investigations in China, the percentage of coal and gas outbursts in coal-mine accidents has steadily increased from 2006 to 2010; more than 2060 casualties have been caused by coal and gas outbursts since 2001^12–14^. Hence, industry and academia are both working to find a reliable technique to predict fracture development in advance.

However, the prediction of fracture development and distribution is difficult due to the heterogeneity of rock formations. There are many methods for predicting fractures based on geological analysis and geophysical logs. Murray *et al*. connected tectonic geological features to fracture development by structural curvature^15^. Because tectonic stress is the main cause of structural fractures, Gao *el al*. predicted fractures based on the numerical simulation of paleotectonic stress^16^, while Ortega *et al*. predicted fracture development using the fracture aperture and density statistics of core data^17^. Prediction using formation micro-resistivity imaging logging and other petrophysical logs in conjunction has shown to be relatively accurate in actual cases^18–20^. However, these methods have only proven to be accurate at a particular depth point and there are limitations to their application to three-dimensional geologic bodies.

To ensure coal mining safety, high-accuracy three-dimensional seismic technology can be applied to determine the distribution of the coal seam and extract information on fracture development^21,22^. Conventional three-dimensional P-wave seismic data interpretation can help to identify large faults. However, in our work area, the azimuth width of seismic acquisition is insufficient; thus, for the P-waves, both the travel time and amplitude information are insensitive to the fracture development^23–25^. Additional P-wave exploration methods, such as AVO (azimuthal amplitude variation with offset) and seismic curvature attribute analysis, have been proposed to satisfy the requirements of fracture prediction^26,27^. With multi-component seismic data, Brodic *et al*. predicted fracture systems by mode-conversions^28^. With the increase in the depth of coal mines, P-wave exploration alone is likely to produce uncertain results due to the increase in complex formation heterogeneity^29^. Recently, multi-component seismic exploration has been undertaken in coal fields. It is possible to predict fracture development through the separation of split PS-waves, which is a more direct method than the conventional P-wave method.

The key process in the separation of split S-waves is the Alford rotation, which can rotate the S1- and S2-waves in from shot-receiver coordinates to their true polarization directions^30,31^. Because the pre-stack PS-wave data always have a low signal-to-noise ratio, this method is more appropriate for post-stack seismic data^31,32^. The PS-wave is generated from a P-wave source that reflects and travels upward as an S-wave. In addition, the S-wave source is more cost-effective and rare compared to the P-wave source, and the PS-waves have been proved effective in the characterization of fractured reservoirs and estimation of fluid^33–35^. Thus, the Alford rotation, which was originally designed for orthogonal pairs of S-wave sources, has been extended to PS-waves recorded as radial components (R-components) and transverse components (T-components)^36^. It has been widely applied in many cases to predict fracture development by the separation of a split S-wave^5,37,38^.

Unlike the reservoirs in oil and gas fields, it is difficult to perform high-precision fracture prediction for coal seams due to their thinness. In this study, for coal seam 13-1 in the Huainan coalfield of China, we performed the separation of split PS-waves to predict fracture development and distribution for the safety evaluation of coal mining. Due to the wavefield mixing of the post-stack R- and T-component data, the post-stack Alford rotation may rotate some mixed waves that should not be rotated. Thus, we only applied the Alford rotation on post-stack R- and T-component sections to derive the fracture azimuths, which were then used for the pre-stack Alford rotation to separate the split PS-waves. Moreover, the fracture parameters including the fracture azimuths and the time delays between S1- and S2-waves were derived. Because the tuning effect of the reflections from the roof and floor of thin coal seam 13-1 reduces the resolution of seismic data, we performed the joint PP- and PS1-wave inversion under the constraints of geophysical logs and rock-physics parameters to derive accurate PS1-wave interlayer travel-times. Thus, we could calculate anisotropy parameters to predict mining areas that may be prone to coal and gas outbursts.

## Geological Setting

**Figure 1:**
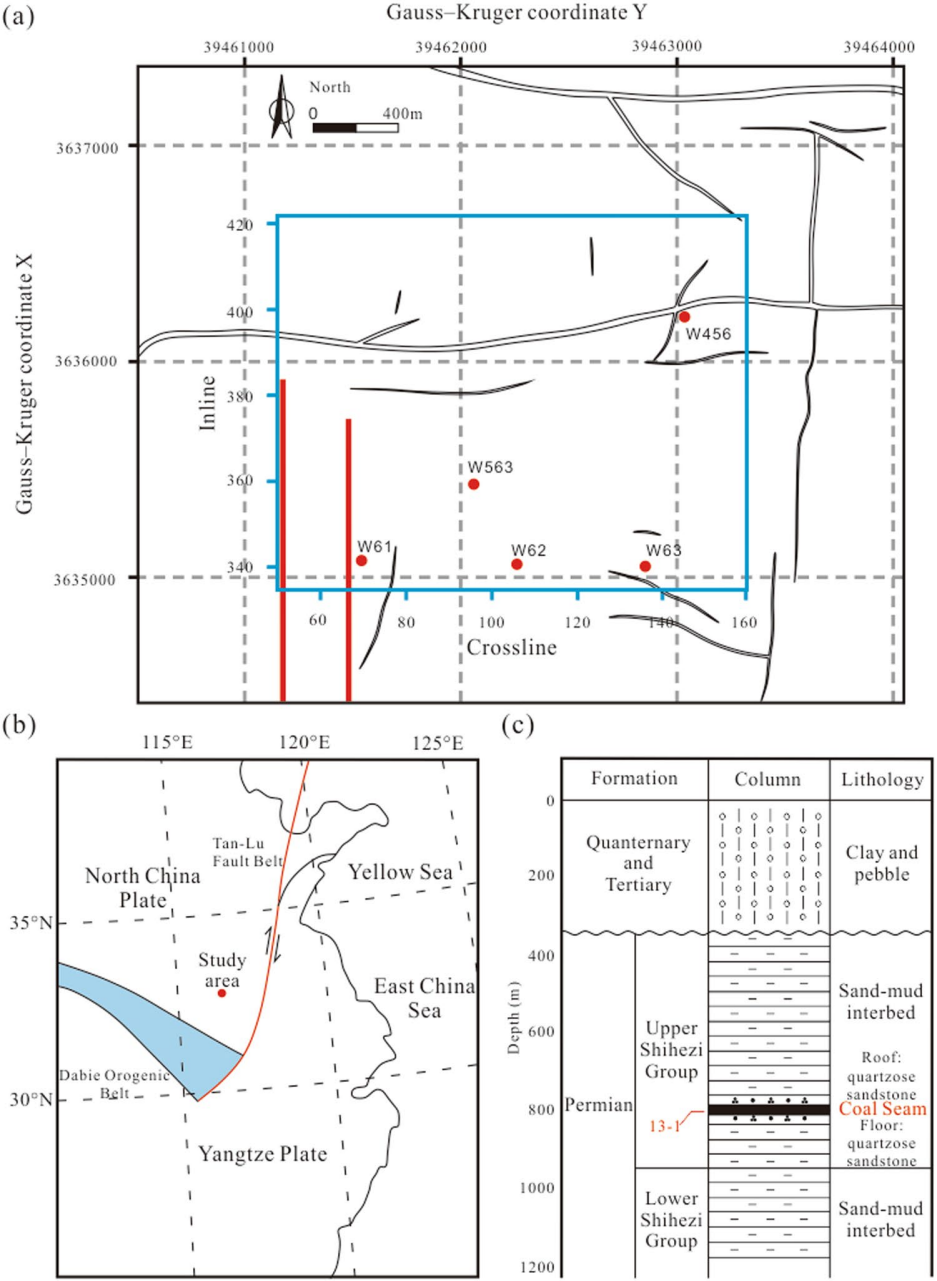
(**a**) Structural sketch map of the Huainan coalfield. The blue rectangle indicates our study area, the two red lines mark two mining tunnels, and the red solid circles mark the well positions. (**b**) Location of the Huainan coalfield (32°51′N, 116°35′E) in the tectonic belts of China. (**c**) Stratigraphy of the Huainan coalfield in well W563. The Gauss–Kruger coordinate system is a projection of the ellipsoid of the earth, which can be converted from latitude and longitude data^50^.

The Huainan coalfield is located at the intersection of the NWW-trending Dabie orogenic belt and the NNE-trending Tan-Lu fault belt. This coalfield belongs to the southern margin of the North China plate, where a large number of faults have developed (Fig. 1a,b). Commercial coal seams in this area, including coal seams 13-1 (target coal seam), 11-2, 8, and 6, developed in the Permian Shihezi Formation. The Tertiary overburden, the lithology of which is dominated by clay gravels, forms an angular unconformity (Fig. 1c). The coal-bearing basal stratum is formed by Paleozoic marine sediments^39^; sandstone and shale alternate between the adjacent coal seams in coal-bearing strata.

After coal accumulation, the coal measures were influenced by the Indo-Chinese and Yanshan orogenies. In the study area, WNW-trending thrust-nappe faults and folds developed in the NS-trending compressional stress field of the Indo-Chinese orogeny. In the Yanshan orogenic movement, the structures formed during the Indo-Chinese orogeny were crosscut by NNE−NE-trending sinistral transpressional faults with a WNW-trending transpressional stress field formed by the EW-trending regional compression and the sinistral shear of the Tan-Lu Fault. Due to these tectonic movements, fractures widely developed in coal seams. However, only the high-angle fractures are retained due to the pressure of overlying strata.

The research target was coal seam 13-1 in the upper Shihezi Formation, in which the fracture development has been revealed by drill cores. Coal seam 13-1 is a typical thin stratum with an average thickness of 4.4 m, and its roof and floor lithology are dominated by medium-fine quartzose sandstone (Fig. 1c). The 13-1 coal samples collected in the mining tunnels show that the high-angle fractures induced by the tectonic stress are larger than the coal cleats (photographs of the 13-1 coal samples are shown in Supplementary Fig. S1).

## Data

The multi-component seismic data used in this study were acquired by the Huainan Coal Mining Group in 2008. As shown in Fig. 1a, the work area marked by the blue solid rectangle contains five wells with acoustic logs, which were transformed to P-wave velocity (*V*_P_) logs. Table 1 shows the main acquisition parameters of the seismic survey in the study area (the distribution of source and receiver points is shown in Supplementary Fig. S3). The azimuth-offset distribution shows an offset within 800 m that can cover all of the observation azimuths and the offset is mainly in the range of 400–800 m (for the Azimuth-offset distribution, see Supplementary Fig. S4). Because the depth of coal seam 13-1 is about 800 m, incidence angles mainly range from 25 to 45°, i.e., small and medium incidence angles.

**Table 1:** Main acquisition parameters of the seismic survey.

Type of seismic acquisition geometry	Block geometries (32 shots and 7 receiver lines per block) (see Supplementary Fig. S2)
Number of multi-component receivers of one shot	7 lines × 30 receivers/line
Source type	Explosive (1.5–2.5 kg, for villages 0.5–1.0 kg)
Source spacing	40 m
Receiver type	3C MEMS
Receiver spacing	40 m
Source line interval	90 m
Receiver line interval	90 m
CDP grid	20 × 20 m^2^
Coverage area	1740 × 2180 m^2^
Sampling rate	1 ms

The seismic data were processed by the Multi-wave and Multi-component (MWMC) Group using the Multi-component Processing System software (see Supplementary Note 1). As shown in Table 2, horizontal rotation was performed to rotate X- and Y-components to R- and T-components. Then, we jointly processed the Z-, R- and T-components. Preserving the polarization direction and amplitude features was the key to ensuring the predictive accuracy of the fracture parameters. Therefore, we used the wave vector characteristics method to suppress noise and surface waves while preserving the amplitude feature^40^. The same parameters of spherical divergence correction were adopted for the R- and T-components to recover the PS amplitudes. Subsequently, we applied the same static corrections to the R- and T-components^41,42^. Thus, we derived the pre-stack seismic data that were used in the later sections. Note that the deconvolution was not included in the data process, because the change of wavelet shape would have affected the prediction of fracture development.

**Table 2:** Processing procedures for three-component pre-stack time migration.

Initial Shot Data	
Horizontal Rotation: X- and Y-components → R- and T-components	
Z-component	R- and T-components
Noise and Surface-wave Suppression	Noise and Surface-wave Suppression
Spherical Divergence Correction	Spherical Divergence Correction
Static Correction	Static Correction
Common-middle-point (CMP) Gather Sorting	Common-conversion-point (CCP) Gather Sorting
P-wave Velocity Analysis	Effective PS Converted-wave (C-wave) Velocity (*V*_C_) Analysis
Generate Common-imaging-point (CIP) Gather	
P-wave Migration Velocity (*V*_P_) Analysis	
Iteratively Generate CIP Gather and Update *V*_P_ until *V*_P_ is Satisfied	
Stretch *V*_P_ from PP Time to PS Time	
	Generate Initial P- to S-wave Migration Velocity Ratio (*λ*_0_) using C-wave Velocity and Stretched *V*_P_ Model: ${\lambda }_{0}=\frac{2{V}_{{\rm{P}}}}{{V}_{{\rm{C}}}}-1$
	Generate CIP Gather
	PS-wave Migration Velocity (*V*_PS_) Analysis
	Iteratively Generate CIP Gather and Update *V*_PS_ until *V*_PS_ is Satisfied, then Calculate Updated P- to S-wave Migration Velocity Ratio (*λ*): $\lambda =\frac{2{V}_{{\rm{P}}}}{{V}_{{\rm{PS}}}}-1$
Pre-stack Time Migration	Pre-stack Time Migration

For the three-component migration, velocity analysis is very important. We sorted Z-component CMP gathers and R- and T-component CCP gathers using the preprocessed shot records. The velocity was analyzed based on the CMP and CCP gathers to derive initial velocity models of P-waves and C-waves, respectively. Then, we performed two loops. For the Z-component, we generated CIP gathers using the initial P-wave velocity model. CIP gather generation and *V*_P_ analysis were performed iteratively until the updated *V*_P_ model was satisfied. To analyze the R- and T-component migration velocity, we first built the initial *λ*_0_ model using the final updated *V*_P_ model stretched from PP time to PS time and a *V*_C_ model. Then, we iteratively generated the PS-wave CIP gathers and updated the *λ* model. Eventually, the pre-stack migration was performed on the three-component seismic data using their corresponding velocity models^43,44^. In post-stack R- and T-component seismic sections, the location of PS-wave reflectors were corrected under the convergence of diffraction waves in migration. The middle part of the study area has good PS-wave fold coverage (up to 350 times), which provided the basis for high-resolution and anisotropy analysis of coal seam 13-1 (for fold coverage, see Supplementary Fig. S5).

## Method

### Separation of split PS-waves

**Figure 2:**
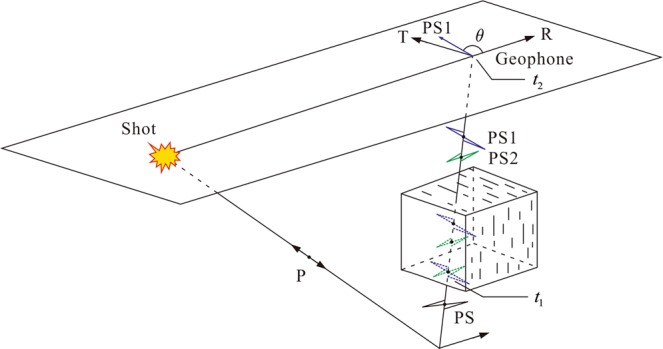
Diagram of PS-wave splitting. The PS-wave is split into PS1- and PS2-waves with polarization directions parallel and orthogonal to the fracture plane, respectively; *θ* is the angle between the R-component direction and the PS1-wave polarization direction.

To predict fracture development, the azimuth and development degree of fracture are the most important parameters. In our method, we use the PS1-wave polarization direction and anisotropy parameter *γ* to describe the fracture azimuth and development degree. As shown in Fig. 2, if a near-surface low-velocity zone exists, PS-waves can be considered to be recorded simultaneously by an R-component with the source-receiver direction and an orthogonal T-component. When the polarization direction of the up-going PS-wave is oblique to the fracture plane, the PS-wave will be split into PS1- and PS2-waves with the polarization directions parallel and orthogonal to the fracture plane, respectively. The PS1-wave travels faster than the PS2-wave in the fractures; thus, at the geophone receiving time *t*_2_, the seismic phases of the PS1- and PS2-waves can be expressed as:

1 $$[\begin{array}{c}\mathrm{PS1}({t}_{2})\\ \mathrm{PS2}({t}_{2})\end{array}]=[\begin{array}{c}\mathrm{PS1}({t}_{1}+\tau /2)\\ \mathrm{PS2}({t}_{1}-\tau /2)\end{array}],$$

where *t*_1_ is the moment of the PS1-wave splitting and *τ* denotes the time delay between the split PS-waves. Then, the R- and T-component data can be described as:

2 $$[\begin{array}{c}{\rm{R}}({t}_{2})\\ {\rm{T}}({t}_{2})\end{array}]=[\begin{array}{cc}\cos \,\theta  & \sin \,\theta \\ \sin \,\theta  & -\cos \,\theta \end{array}]\,[\begin{array}{c}\mathrm{PS1}({t}_{1}+\tau /2)\\ \mathrm{PS2}({t}_{1}-\tau /2)\end{array}],$$

where *θ* denotes the angle between the R-component direction and the PS1-wave polarization direction.

In summary, the R- and T-component seismic data can be considered as the mixing of the PS1- and PS2-waves containing fracture information. Because the PS1-wave polarization direction indicates the fracture azimuth, we could determine the fracture azimuth by searching for the PS1-wave polarization direction. The degree of fracture development can be quantitatively predicted by the anisotropy parameter. We can derive the anisotropy parameter *γ* as^37^:

3 $$\gamma =\frac{\tau }{{\rm{\Delta }}{t}_{{\rm{PS}}1}},$$

which reflects the intensity of the fracture development within the target stratum. The parameter *τ* denotes the time delay between the split PS-waves within coal seam in the vertical direction, and Δ*t*_PS1_ signifies the interlayer travel-time of the PS1-wave between the top and bottom interfaces of coal seam 13-1 (see Supplementary Fig. S6).

We used a two-step Alford rotation approach to separate the split PS-waves. First, we applied the Alford rotation to the post-stack R- and T-component data by:

4 $$[\begin{array}{c}{\rm{PS1}}({t}_{1}+\tau /2)\\ {\rm{PS2}}({t}_{1}-\tau /2)\end{array}]=[\begin{array}{cc}\cos \,\theta  & \sin \,\theta \\ \sin \,\theta  & -\cos \,\theta \end{array}]\,[\begin{array}{c}{\rm{R}}({t}_{2})\\ {\rm{T}}({t}_{2})\end{array}],$$

where the rotation angle *θ* is determined by scanning a series of angles *b* from 0 to 180° using:

5 $$[\begin{array}{c}{{\rm{PS1}}}^{\ast }({t}_{1}+\tau /2)\\ {{\rm{PS2}}}^{\ast }({t}_{1}-\tau /2)\end{array}]=[\begin{array}{cc}\cos \,b & \sin \,b\\ \sin \,b & -\cos \,b\end{array}]\,[\begin{array}{c}{\rm{R}}({t}_{{\rm{2}}})\\ {\rm{T}}({t}_{{\rm{2}}})\end{array}],$$

where the asterisk denotes the intermediate result of the split PS-wave separation. Only when:

6 $$\tan \,b=\frac{{{\rm{PS2}}}^{\ast }({t}_{1}-\tau /2)}{{{\rm{PS1}}}^{\ast }({t}_{1}+\tau /2)}$$

and *b* are equal to the fracture azimuth *θ*, are the split PS-waves completely separated. The time delay *τ* is then derived by tracing the separated PS1- and PS2-wave reflection times.

Normally, the PS1- and PS2-waves split from the target stratum are multi-split, because fractures of different azimuths and scales are developed in different layers of the overlying strata. We used the layer stripping approach with a sliding time window to separate the split PS-waves from the overlying strata to the target stratum. The fracture azimuths scanned from first time window by the Alford rotation are adopted to rotate all the R- and T-component data below this time window. Then, at the next time window, we performed the same Alford rotation and data rotation. Thus, the separated PS1- and PS2-waves of the target stratum preserved the accumulation of the time delays from all the preceding time windows (see Supplementary Fig. S7). In many cases, non-vertical fractures also develop in strata. For this geological model, the Z-component is required to separate split shear waves (see Supplementary Note 2 and Supplementary Fig. S8). However, in our work area, only X- and Y-components were recorded for the PS-wave reflections of coal seam 13-1 (marked by the red dashed curves in Supplementary Fig. S9). Thus, no PS-wave projections of coal seam 13-1 on the Z-component are available. Therefore, the fractures developed in coal seam 13-1 are mainly vertical.

### Test of the theoretical model

**Figure 3:**
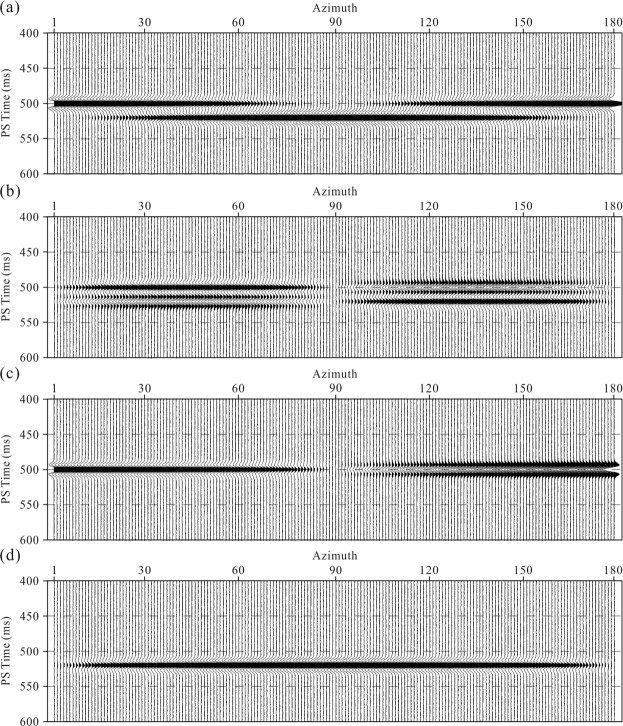
(**a**,**b**) Synthetic R- and T-component azimuth sections, respectively. (**c**,**d**) Separated PS1- and PS2-wave azimuth sections using the Alford rotation, respectively.

The Alford rotation is a method for recovering the PS1- and PS2-wave polarizations that contain fracture information. As shown in Fig. 3, we tested the performance of the Alford rotation using synthetic data. The work flow for the fracture orientation scanning are as follows:

1. Simulate the pure PS1- and PS2-waves with a 20-ms time delay. Assume 0–180° angles between the R-component and PS1-wave polarization directions, and derive the R- and T-component data by the rotation according to equation (2). Then, add random noise of 10% level into the R- and T-components (Fig. 3a,b, respectively).
2. Two-step Alford rotation scanning to derive the fracture azimuths using equations (4–6). Separate the PS1- and PS2-waves by the Alford rotation of the synthetic R- and T-components using calculated fracture azimuths (Fig. 3c,d, respectively).

As shown in Fig. 3a,b, the events of the R-component azimuth section present a “sinusoidal” shape and a clear azimuth time delay. In the T-component azimuth section, when the T-component azimuths are parallel or orthogonal to the fracture plane, the energy is approaches zero, and the polarity reverses. When the fracture azimuths are equal to 0° or 90°, the R-component azimuth is parallel or orthogonal to the fracture plane; therefore, as shown in Fig. 3c,d, the energy of PS2- or PS1-wave section is close to zero. We can see that even under noisy conditions, the fracture azimuth and time delay derived from the Alford rotation are close to the true values. The fracture azimuths can be derived by angle scanning; however, the precision of the time delay tracing depends on the quality of the seismic imaging.

## Results and Discussion

### Inversion of PS1-wave interlayer travel-time

To derive the anisotropy parameter *γ* in equation (3), we derived Δ*t*_PS1_ by summing the one-way interlayer travel-time of down-going P-waves and up-going PS1-waves

7 $${\rm{\Delta }}{t}_{{\rm{PS1}}}=\frac{{\rm{\Delta }}{t}_{{\rm{PP}}}}{2}+\frac{{\rm{\Delta }}{t}_{{\rm{SS}}}}{2},$$

where Δ*t*_PP_ and Δ*t*_SS_ are the PP-wave and SS-wave two-way interlayer travel-time of coal seam 13-1, respectively, and

8 $$\begin{array}{rcl}{\rm{\Delta }}{t}_{{\rm{PP}}} & = & \frac{2h}{{V}_{{\rm{P}}}},\\ {\rm{\Delta }}{t}_{{\rm{SS}}} & = & \frac{2h}{{V}_{{\rm{S}}}},\end{array}$$

where, *h* denotes the thickness of coal seam 13-1, *V*_P_ and *V*_S_ are the P- and S-wave velocities, respectively. Here, the S-wave specifically refers to the S1-wave. We defined *λ* as the P- to S-wave velocity ratio

9 $$\lambda =\frac{{V}_{{\rm{P}}}}{{V}_{{\rm{S}}}}.$$

Substituting equations (8) and (9) into equation (7), we obtain

10 $${\rm{\Delta }}{t}_{{\rm{PS1}}}=\frac{{\rm{\Delta }}{t}_{{\rm{PP}}}}{2}+\lambda \frac{{\rm{\Delta }}{t}_{{\rm{PP}}}}{2}.$$

Due to the tuning effect, the top and bottom interfaces of coal seam 13-1 could hardly be distinguished on either the PP- or PS1-wave post-stack section. Thus, we performed the PP- and PS1-wave joint inversion to improve the resolution of the top and bottom interfaces and derived *λ* under the well constraints.

Ultrasonic measurements of drill cores by Lu *et al*. indicate an empirical correlation between the P- and S-wave velocities of the sand and mudstone strata in our study area:

11 $${V}_{{\rm{S}}}=0.433{V}_{{\rm{P}}}+430.9,$$

which was adopted to transform the P-wave acoustic logs to the S-wave acoustic logs^11^. For the transformation of the S-wave log in coal seam 13-1, we adopted the empirical correlation proposed by Wang *et al*.^45,46^:

12 $${V}_{{\rm{S}}}=0.5208{V}_{{\rm{P}}}+\mathrm{110.67.}$$

Because there are no empirical correlations for the transformation of the density logs in the study area, we adopted the Gardner equation for the sand and mudstone strata^47^:

13 $$\rho =0.31{V}_{{\rm{P}}}^{0.25},$$

which is widely applied to sedimentary strata^44^. We applied the empirical formula given by Wang *et al*.^45,46^ to coal seam 13-1:

14 $$\rho =1.85-\frac{648.15}{{V}_{{\rm{P}}}}.$$

**Figure 4:**
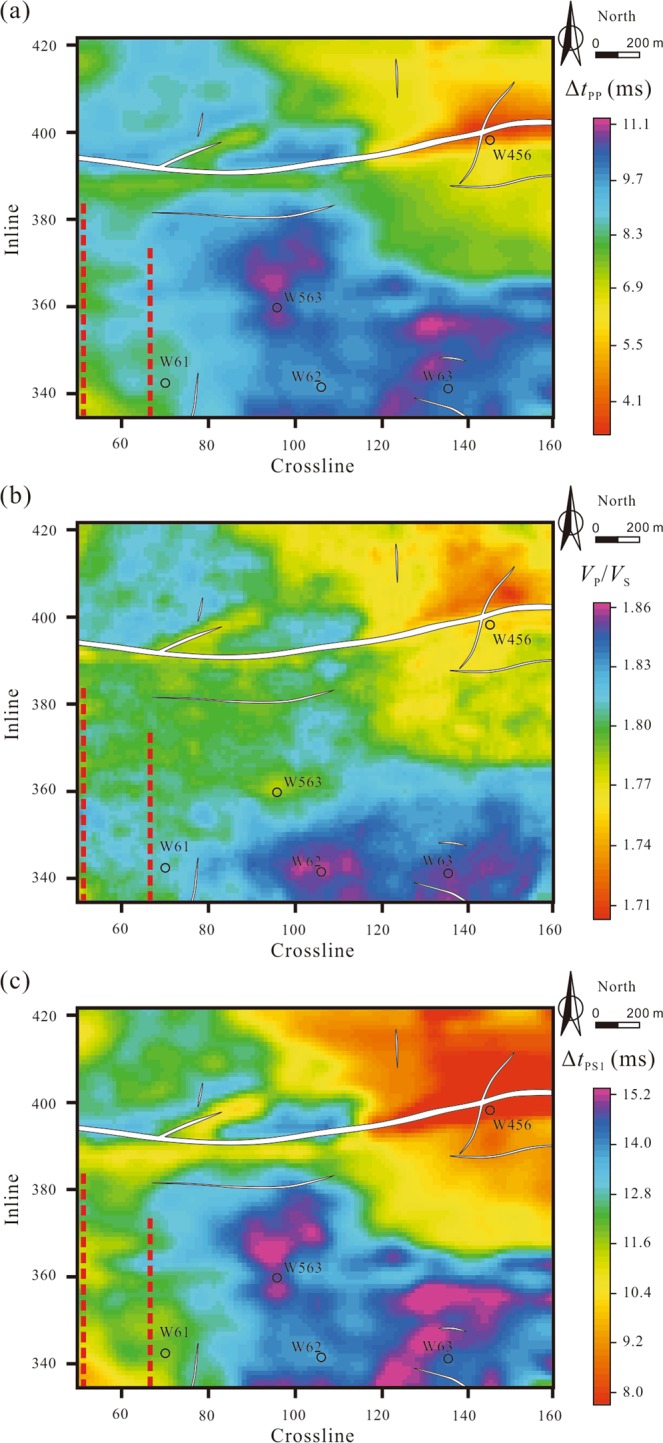
Joint inversion results of PP- and PS1-waves. (**a**) PP-wave interlayer travel-time of coal seam 13-1; (**b**) P- to S-wave velocity ratios of coal seam 13-1; (**c**) PS1-wave interlayer travel-time of coal seam 13-1. The two red dotted lines mark two mining tunnels.

We used the PP- and PS1-wave AVO gathers compressed to the PP time to invert the P- and S-wave velocities (*V*_P_ and *V*_S_) and density (*ρ*) sections based on the models interpolated using the well logs^48^. Then, the P-wave impedance sections (see Supplementary Fig. S10) can be calculated by *V*_P_ and *ρ*, and Δ*t*_PP_ can be derived from the top and bottom interfaces on the impedance sections (Fig. 4a). With the parameter *λ* derived from *V*_P_ and *V*_S_, we transformed Δ*t*_PP_ to Δ*t*_PS1_ according to equation (7) (Fig. 4b,c).

### Results of Alford rotation

**Figure 5:**
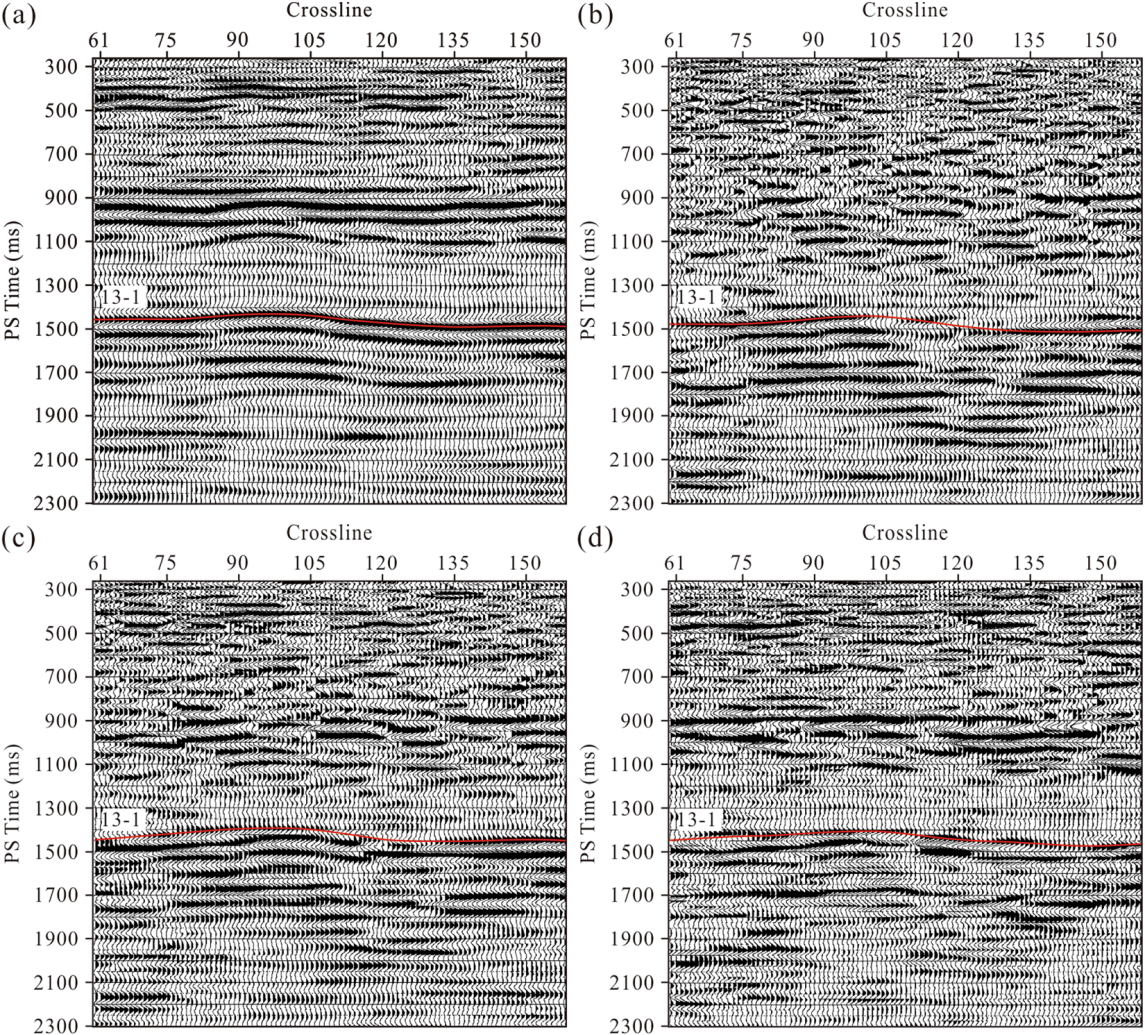
Results of the Alford rotation using the post-stack sections at inline 357: (**a**) R-component, (**b**) T-component, (**c**) PS1-waves, and (**d**) PS2-waves. The red line shows the interpreted travel-time for the 13-1 coal seam.

**Figure 6:**
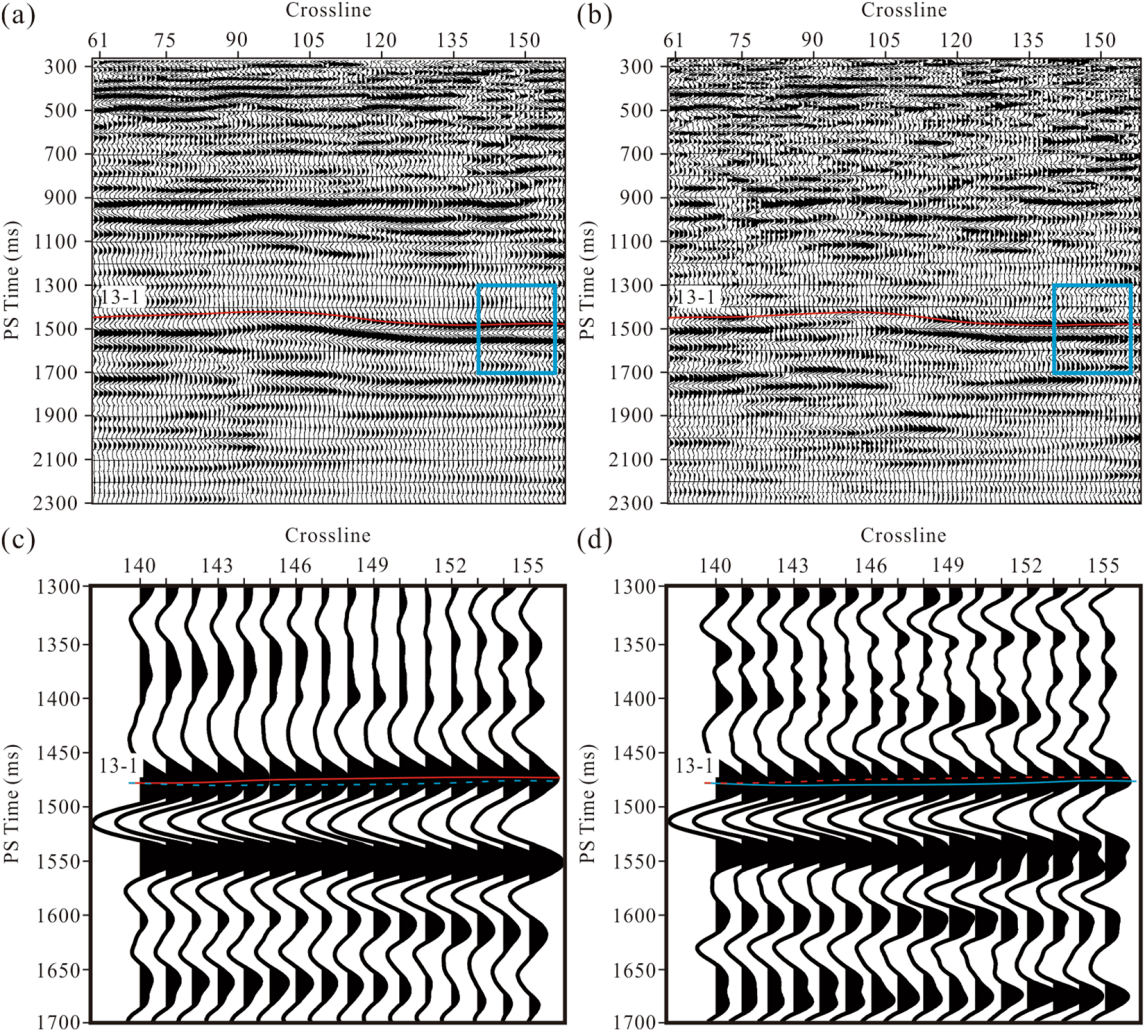
Results of the Alford rotation using pre-stack seismic data at inline 357: (**a**) PS1-waves, and (**b**) PS2-waves. The rotation angles are scanned based on the R- and T-component post-stack sections. The PS1 (**a**) and PS2 (**b**) events in the blue rectangular frames are partially enlarged in (**c**) and (**d**), respectively. The red and blue lines indicate the PS1- and PS2-wave horizons of coal seam 13-1, respectively.

We applied the Alford rotation to the migrated R- and T-components of the study area. As displayed in Fig. 5a,b, the R- and T-component events do not reflect the true formation occurrences due to the wavefield mixing caused by the shear wave splitting. Compared with post-stack seismic data, pre-stack seismic data have a relatively low signal-to-noise ratio and can hardly be used to calculate fracture azimuths. Therefore, we choose to use post-stack data with a high signal-to-noise ratio to calculate the fracture azimuth. However, during the process of pre-stack time migration, the wavefield at each imaging bin is stacked by the amplitudes at different offsets. Thus, the fracture azimuth for each bin represents a dominant azimuth. Therefore, when we used the azimuths to rotate the post-stack data, some stacked waves can hardly be rotated to the real directions. We subsequently used the layer stripping approach to separate the PS1- and PS2-waves with a sliding time window of 30 ms. Although there are thin fractured strata above coal seam 13-1, the fracture azimuths of this coal seam can be derived directly based on layer stripping. The results of the Alford rotation in Fig. 5c,d show that time delays between the PS1- and PS2-waves exist in coal seam 13-1. However, it is inaccurate to trace the time delays due to the wavefield mixing. Therefore, in this method, we used only the fracture azimuths derived from the post-stack data to rotate the pre-stack R- and T-component records, which were then migrated to be the PS1- and PS2-wave sections (Fig. 6a,b). Compared with the post-stack rotated sections in Fig. 5c,d, the seismic events in Fig. 6a,b are clearer and more stable. Wavefield mixing has less influence on the split PS-wave separation in this method. The events marked by the blue rectangular frames in Fig. 6a,b are partially enlarged and shown in Fig. 6c,d, respectively, where clearer and more accurate time delays can be seen between the PS1- and PS2-waves indicated distinctly by the PS1-wave (red line) and PS2-wave (blue line) horizons.

### Prediction of fracture development

**Figure 7:**
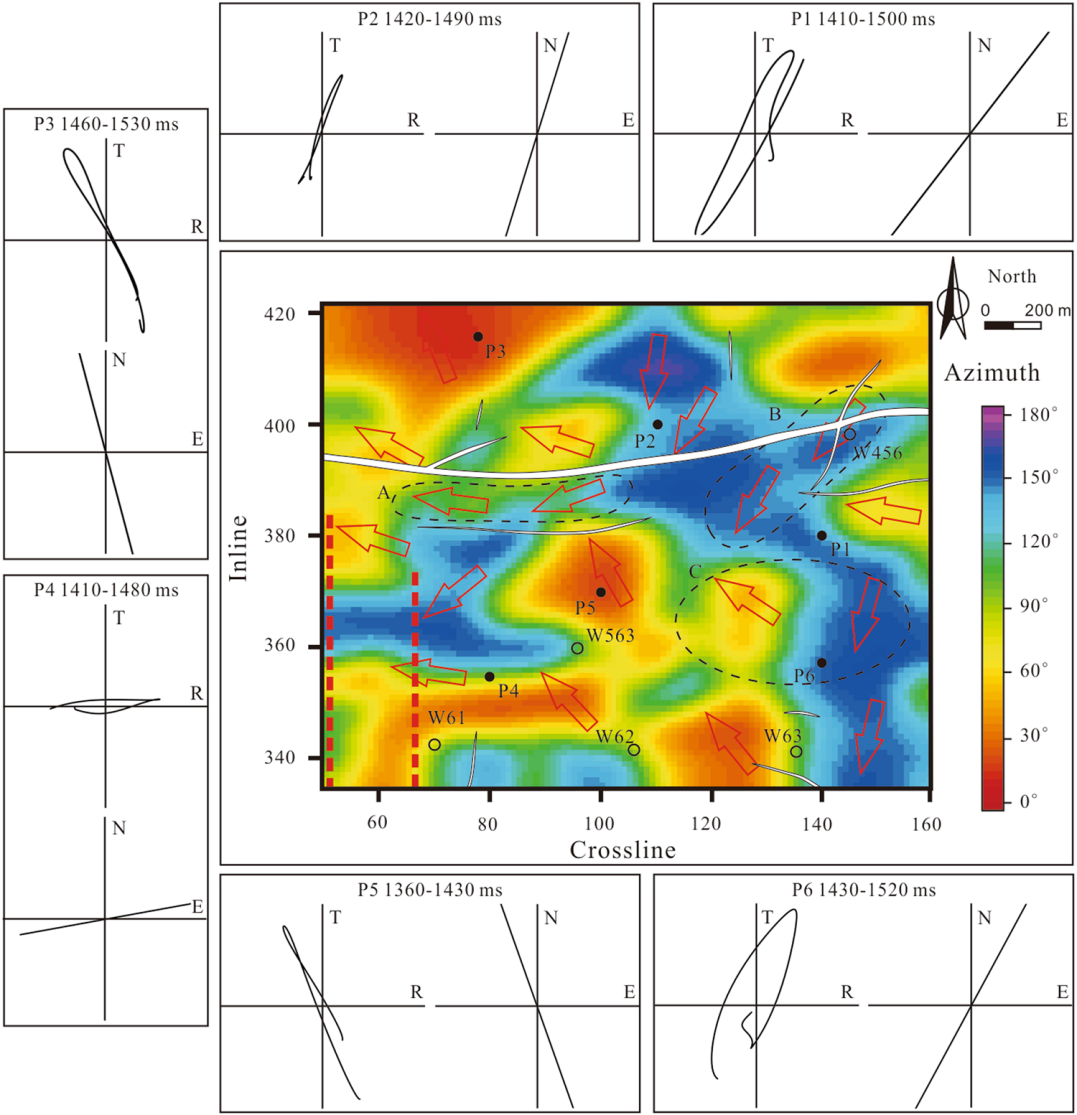
Fracture azimuths of coal seam 13-1. Red arrows indicate the primary fracture azimuths. Contrasts between particle motion and the results of fracture azimuth prediction at the windows of coal seam 13-1 are also shown (R: radial component; T: transverse component; N: north direction; E: east direction).

**Figure 8:**
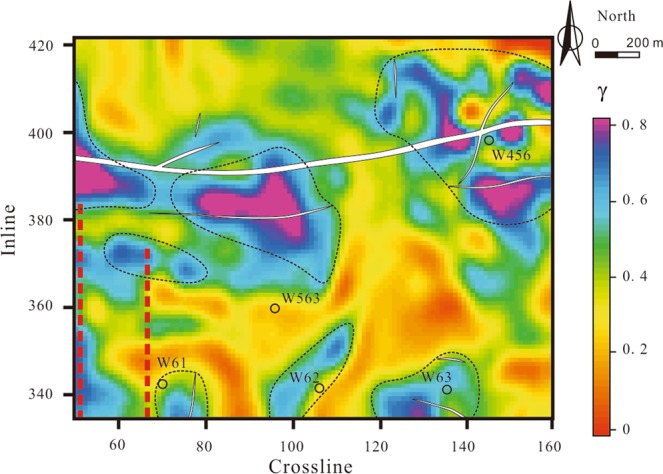
Anisotropy parameters induced by the fractures developed in coal seam 13-1. Red arrows indicate the primary fracture azimuths. Black dotted curves enclose the unsafe mining areas, where the fractures are strongly developed.

The fracture azimuths obtained from the Alford rotation of the post-stack data are shown in Fig. 7. The fracture azimuths are consistent with particle motion. In areas around an isolated fault, such as region A in Fig. 7, the fracture azimuths are basically consistent with the fault strike due to the single stress direction. In areas around intersecting faults, such as region B in Fig. 7, the fracture azimuths are affected by several stress directions. Therefore, the fracture azimuths are less consistent with the fault strikes. In areas far away from the faults (region C in Fig. 7), we cannot easily observe a relationship between the fracture azimuth and a particular fault strike. In these areas, various factors can lead to the formation of fractures, such as stratum shrinkage and local tectonic stress^49^. We extracted the time delays between coal seam 13-1 picked on pre-stack migrated PS1- and PS2-waves; combined with Δ*t*_PS1_ derived from the PS1-waves inversion, we derived the anisotropy parameters (*γ*) of coal seam 13-1 by equation (3). Before extracting the time delays, we did not performed deconvolution to both the R- or T-component data, because doing so would change the waveforms of wavelets and affect the calculated time delays of split shear waves. However, the anisotropy parameter is the ratio of the time delay to interlayer travel-time, which is less influenced by the deconvolution. Figure 8 shows that the anisotropy near the fault zone and wells W61, W62, and W63 is relatively strong. Therefore, the fractures in these areas are relatively well developed, which has been confirmed by the borehole data provided by Huainan Coal Mining Group. At well W456, the anisotropy parameter is high because the multi-phase structural movements have caused coal seam 13-1 to be seriously eroded and powdered. Fracture development may lead to mining disasters such as coal and gas outbursts and water influx. We predicted some unsafe mining areas (marked by the dashed curves in Fig. 8) close to faults in accordance with the fracture-induced anisotropy parameters.

## Conclusions

We developed a combined method using the pre-stack Alford rotation and joint inversion for the fracture system prediction of coal seams. The results of the theoretical model tested in noisy conditions prove the accuracy of the Alford rotation. Due to the wavefield mixing, we applied the fracture azimuths derived from the post-stack Alford rotation to the pre-stack Alford rotation. To improve the resolution of the anisotropy parameters of the thin coal bed, we adopted the joint inversion of PP- and PS1-waves, and split PS-wave horizon tracing based on the pre-stack migrated real sections.

For field data, the application results of our method are consistent with the prior knowledge of the faults, mining tunnels, and borehole data. Our method has been validated for the prediction of the fracture development and distribution. In addition, our method can be adopted to determine areas with a high possibility of coal and gas outbursts. Our method has the potential to be a practical and feasible technique for predicting coal mine disasters.

## Supplementary information

Supplementary Information
