# Supplementary material for: Prediction of Fractures in Coal Seams with Multi-component Seismic Data
Source: Sci Rep. 2019 Apr 24;9:6488. doi: 10.1038/s41598-019-42956-7 (PMC6482175; doi:10.1038/s41598-019-42956-7)
Supplement: Supplementary file 1 — Supplementary Information [file 41598_2019_42956_MOESM1_ESM.docx]

**Prediction of Fractures in Coal Seams with Multi-component Seismic Data**

Mengqi Li, Jun Lu & Shu Xiong

School of Energy Resources, China University of Geosciences, Beijing, 100083, China

Correspondence and requests for materials should be addressed to J.L. (email: lj615@cugb.edu.cn)

**Supplementary information**

**Note 1**

The McPS is developed by our MWMC Group and available at State Key Laboratory of Geological Processes and Mineral Resources, China University of Geosciences, Beijing and the Department of Geosciences, the University of Tulsa (<https://engineering.utulsa.edu/news/mcps-software-donation/>).

**Note 2**

“To a model with arbitrary dip fracture, as shown in Supplementary Fig. S8, the PS-wave split into PS1- and PS2-waves at the fracture surface at time *t*_1_

$$\left[\begin{matrix} PS1(t_{1}) \\ PS2(t_{1}) \end{matrix}\right]=\left[\begin{matrix} \text{cos}\theta\\ \text{sin}\theta\end{matrix}\right]\text{PS}(t_{1}),$$

where *θ* denotes dips of fracture surface. We assumed that the time delay between the split PS-waves is *τ*. At the geophone receiving time *t*_2_, the R- and Z-component data can be expressed as:

Then, the equation for the split shear wave separation can be rewritten as:

We can find that the form of this equation is same as that of equation (4) in our paper. The only difference is that the T-component in equation (4) of our paper is replaced by a Z-component. Then, we can calculate the fracture dips using the same flow introduced in our paper. When the fracture is vertical, the projection of split shear waves on the Z-component is zero according to equation (3).

**
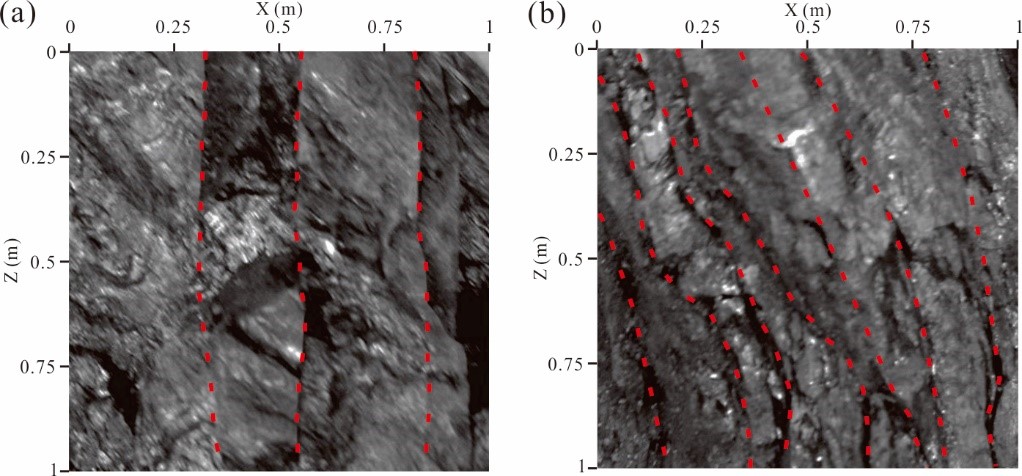
**

**Supplementary Figure S1.** Photographs of the 13-1 coal samples collected in the mining tunnels. Red dotted lines indicate the macroscopic tectonic fractures.


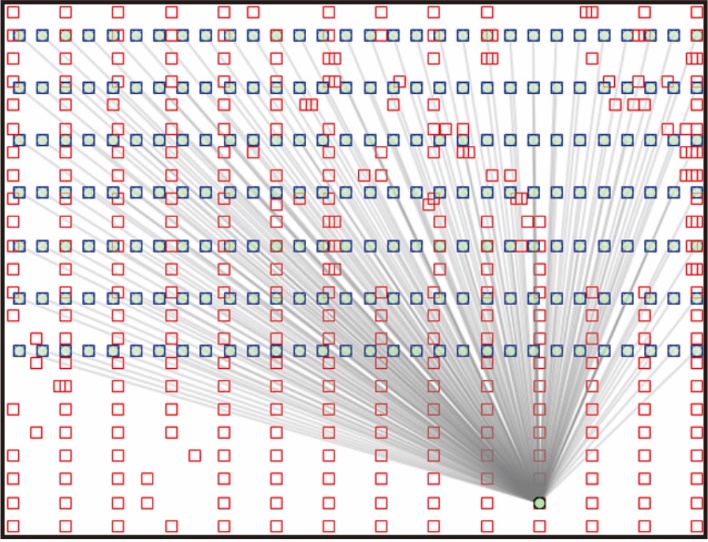


**Supplementary Figure S2.** Sketch of the one shots in a block survey. When a block survey is finished, moving the block at a certain distance to finish the multi-block survey. Blue rectangles indicate receiver points.


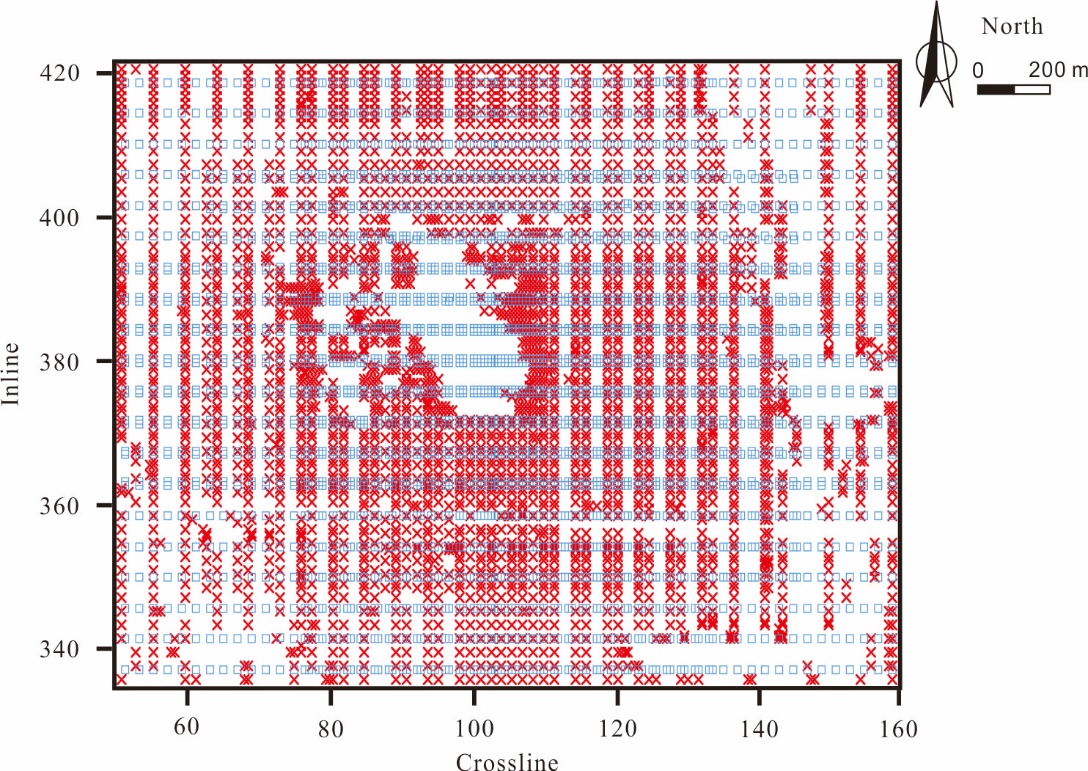


**Supplementary Figure S3.** Distribution of source and receiver points. Red crosses indicate source points, and blue rectangles indicate receiver points.


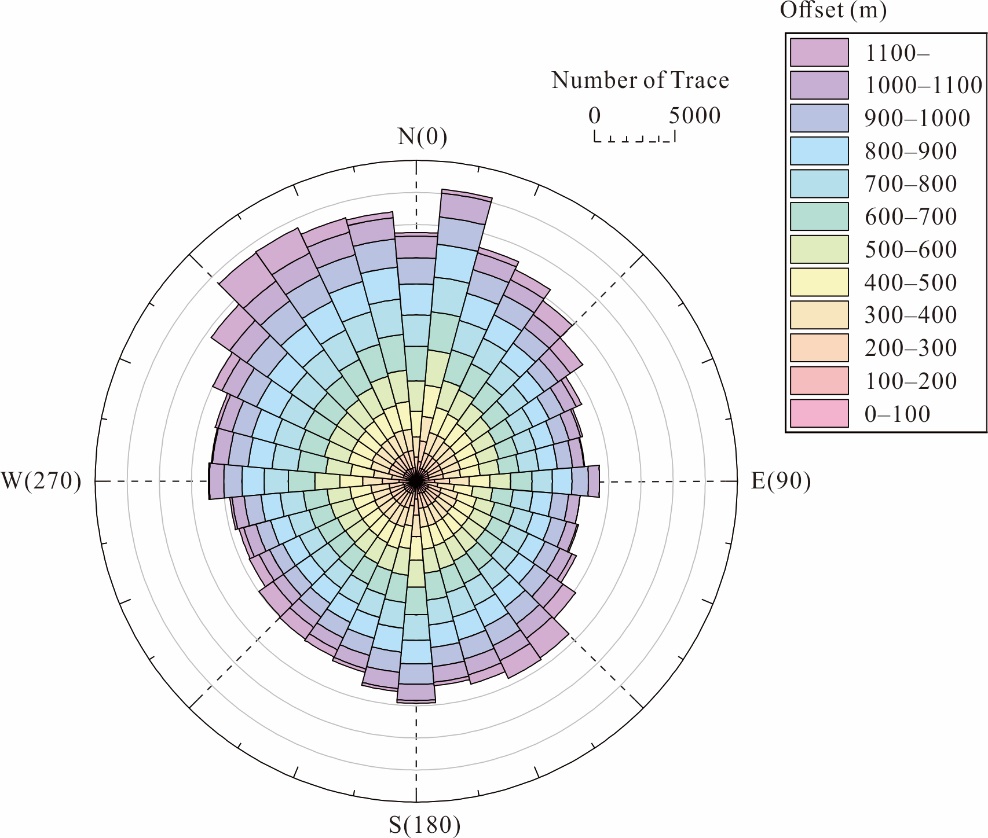


**Supplementary Figure S4.** Azimuth-offset distribution of the seismic data.


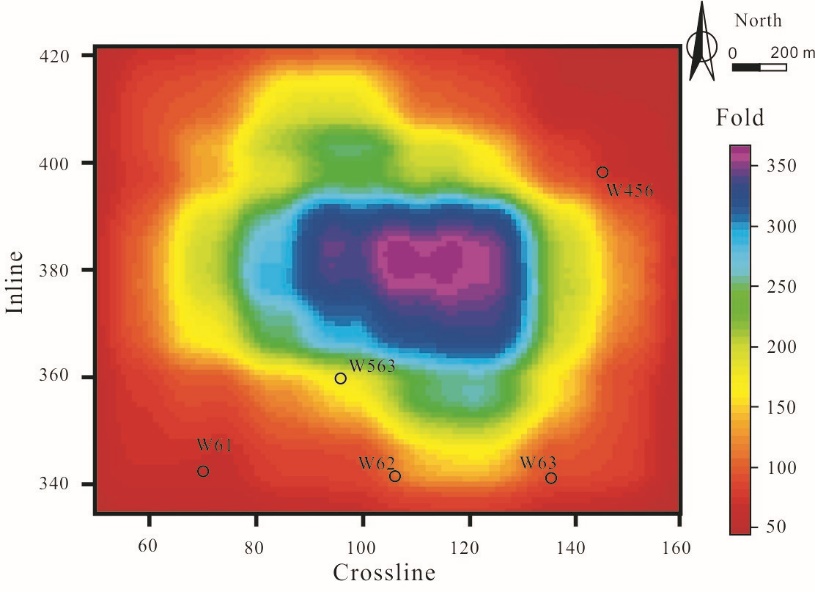


**Supplementary Figure S5.** Fold coverage of the PS-waves.


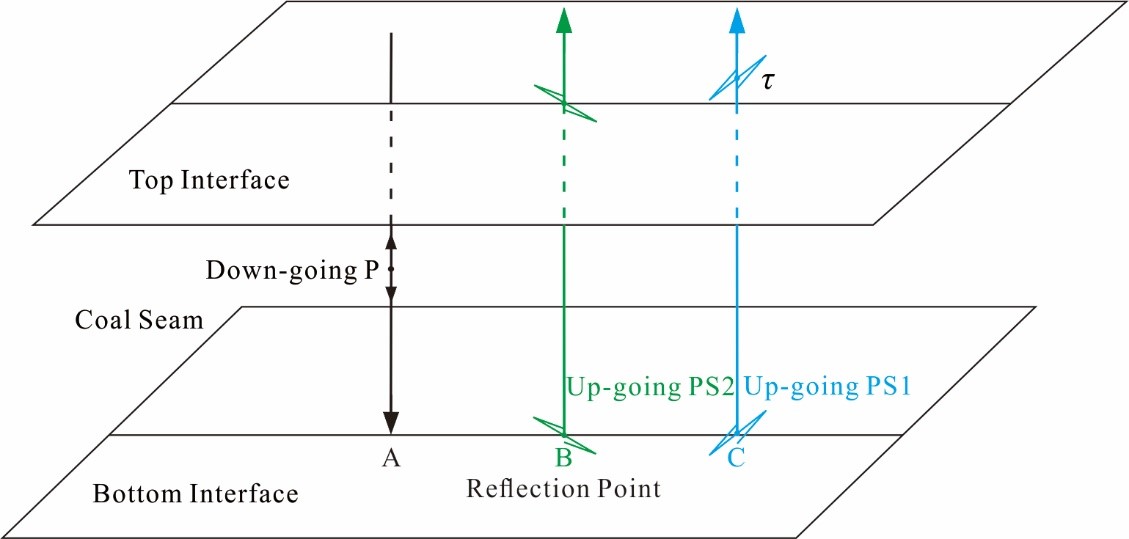


**Supplementary Figure S6.** Relationship between down-going P-wave, up-going PS1-wave and up-going PS2-wave in the vertical direction. Reflection points A, B, and C are at the same point.


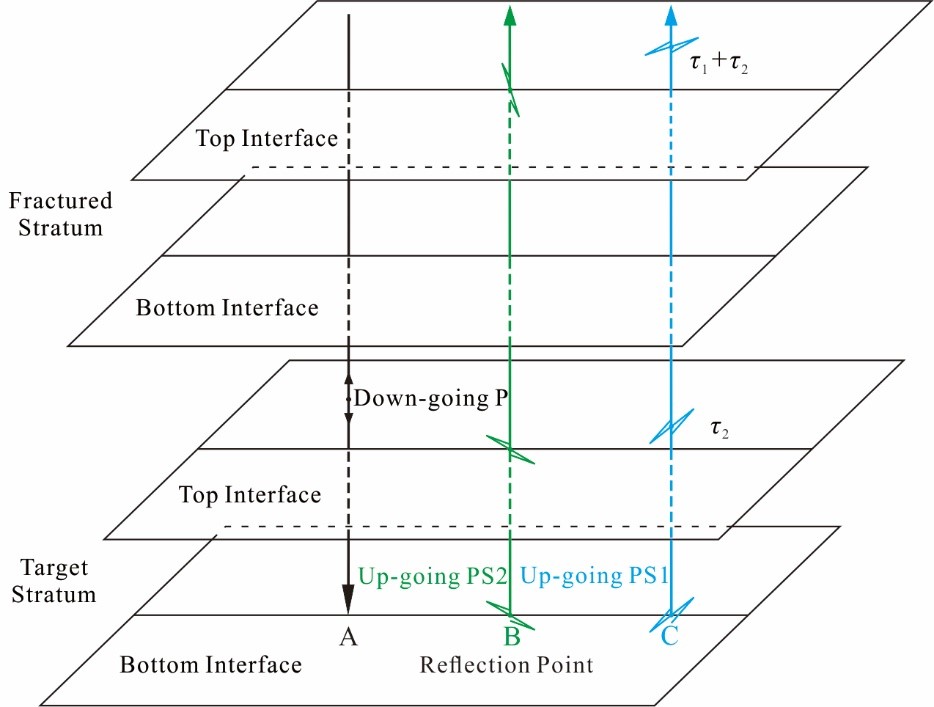


**Supplementary Figure S7.** Sketch of the multi-split PS-waves. Reflection points A, B, and C are at the same point; *τ*_1_ + *τ*_2_ is the time delay recorded on the ground, which contains the time delays *τ*_1_ and *τ*_2_ generated in the upper and target fractured strata, respectively. To derive the time delay of the target stratum, we removed the time delays of the upper fractured strata.


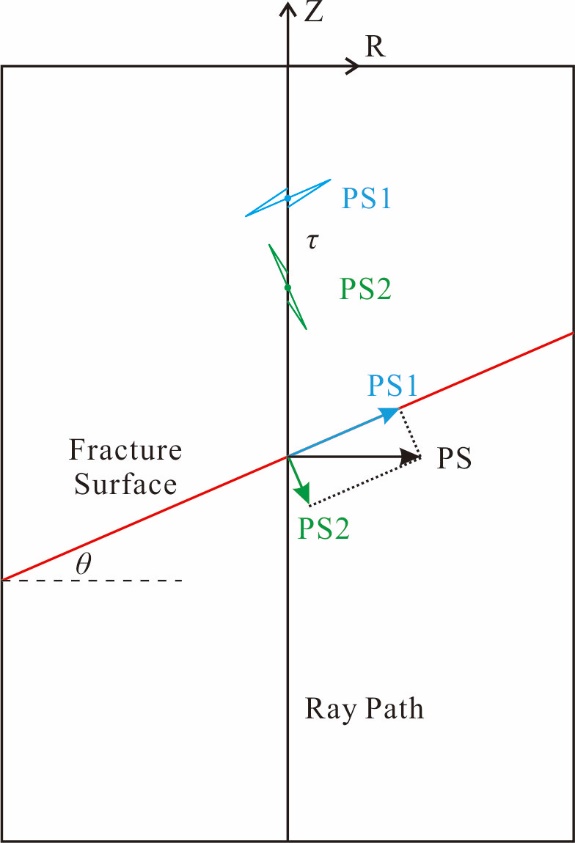


**Supplementary Figure S8.** Diagram of PS-wave splitting of fracture with arbitrary dips. The red line indicates the fracture surface. The black line in the middle indicates the ray path. The PS1- and PS2-waves are marked as blue and green polarization, respectively.


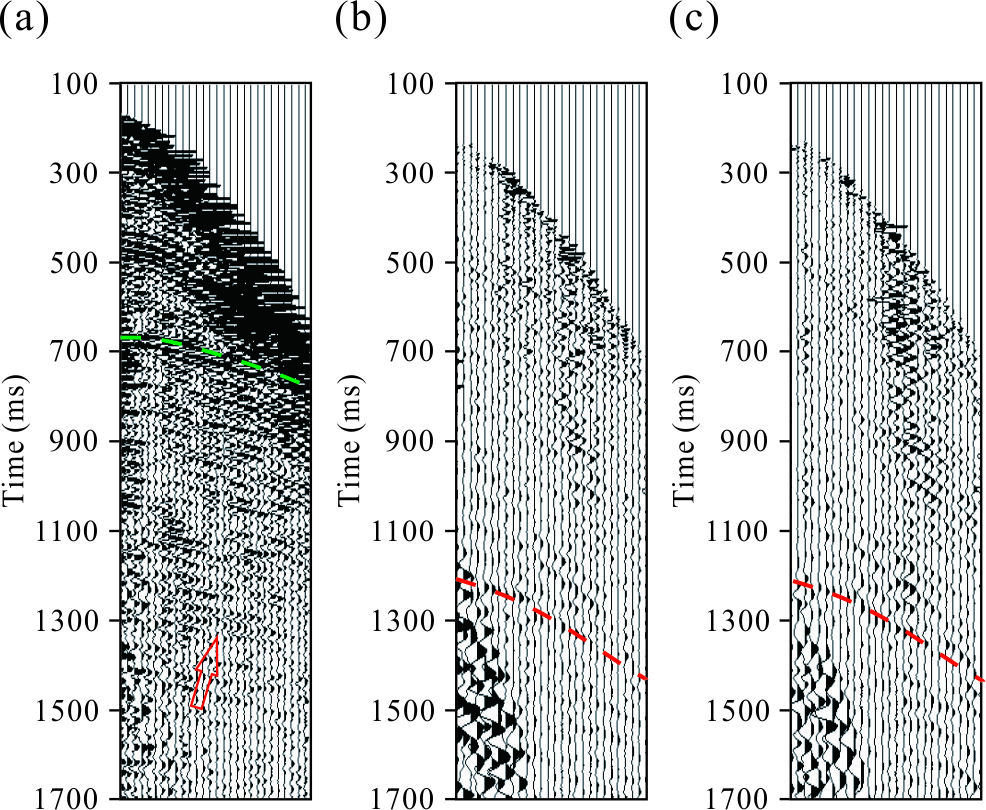


**Supplementary Figure S9.** Initial shot records of Z-component (**a**), X-component (**b**) and Y-component (**c**). Green and red dashed curves mark the PP- and PS-wave reflections of coal seam 13-1, respectively.


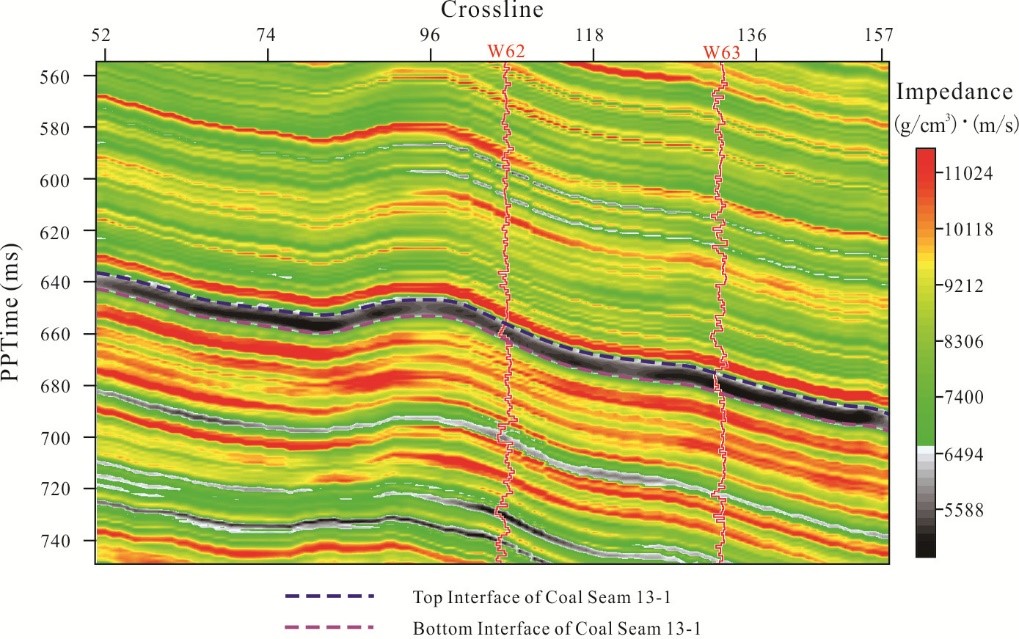


**Supplementary Figure S10.** Inverted P-wave impedance section at inline 341 through wells W62 and W63. Blue and purple dotted lines indicate the top and bottom interfaces of coal seam 13-1.
